# The Use of Antiepileptic Drugs (AEDs) for the Treatment of Pediatric Aggression and Mood Disorders

**DOI:** 10.3390/ph3092986

**Published:** 2010-09-10

**Authors:** Kaizad R. Munshi, Tanya Oken, Danielle J. Guild, Harsh K. Trivedi, Betty C. Wang, Peter Ducharme, Joseph Gonzalez-Heydrich

**Affiliations:** 1 Department of Psychiatry, Children’s Hospital Boston, Harvard Medical School, 300 Longwood Avenue, Boston, MA 02115, USA; 2 Vanderbilt Psychiatric Hospital, Vanderbilt University School of Medicine, 1601 23^rd^ Avenue South, Room 1157, Nashville, TN 37212, USA; 3 Massachusetts General Hospital, Simches Research Building, Harvard Medical School, 185 Cambridge Street, 2^nd^ Floor, Boston, MA 02114, USA

**Keywords:** aggression, anti-epileptic drugs (AEDs), pediatric bipolar disorder

## Abstract

Aggressive symptomatology presents across multiple psychiatric, developmental, neurological and behavioral disorders, complicating the diagnosis and treatment of the underlying pathology. Anti-Epileptic Drugs (AEDs) have become an appealing alternative in the treatment of aggression, mood lability and impulsivity in adult and pediatric populations, although few controlled trials have explored their efficacy in treating pediatric populations. This review of the literature synthesizes the available data on ten AEDs—valproate, carbamazepine, oxcarbazepine, phenytoin, lamotrigine, topiramate, levetiracetam, zonisamide, gabapentin and tiagabine—in an attempt to assess evidence for the efficacy of AEDs in the treatment of aggression in pediatric populations. Our review revealed modest evidence that some of the AEDs produced improvement in pediatric aggression, but controlled trials in pediatric bipolar disorder have not been promising. Valproate is the best supported AED for aggression and should be considered as a first line of treatment. When monotherapy is insufficient, combining an AED with either lithium or an atypical anti-psychotic can result in better efficacy. Additionally, our review indicates that medications with predominately GABA-ergic mechanisms of action are not effective in treating aggression, and medications which decrease glutaminergic transmission tended to have more cognitive adverse effects. Agents with multiple mechanisms of action may be more effective.

## 1. Introduction

Aggression is not in itself a syndrome; it is a non-pathognomonic symptom that represents a common phenotype to multiple psychiatric illnesses (Table 1). Characteristics of aggression implicate different etiologies. Consequently, there is a difference in treating the aggression of an adolescent male experiencing his first psychotic break compared with that of a latency age female with a history of physical and sexual abuse, showing clinically significant anger and irritability towards others. The underlying pathology can be inferred from the severity and duration of the aggressive episode (Table 1). The appropriate diagnosis and treatment of aggression in children and adolescents presents a unique dilemma in clinical care [1]. 

Defining diagnostic categories for the varied types of aggression remains one of the more controversial issues within the field of pediatric mental health. By the Diagnostic and Statistical Manual of Mental Disorders’ (DSM-IV) [2] criteria, most children presenting with severe mood dysregulation meet for either the Attention Deficit Hyperactivity Disorder (ADHD) spectrum disorders implicated by short aggressive outbursts, or mood disorders implicated by more prolonged episodes. The current debate dictating future changes to the DSM centers on the argument that the existing diagnostic structure precludes adequate diagnosing of children presenting with mood dysregulation that is not accounted for by Bipolar or ADHD spectrum diagnoses [3]. As a result, a new diagnosis of Temper Dysregulation Disorder with Dysphoria (TDD) has been proposed for the upcoming DSM-V to create a more accurate diagnostic construct for aggression [3]. The defining symptoms of this disorder include at least 12 months (with remittance no longer than three months) of severe temper outbursts that occur an average of three times a week in response to common stressors which manifest verbally or behaviorally and are disproportionate to the event as well as developmentally inappropriate [4]. The onset of the disorder should occur before 10 years of age, and is defined by persistent negative emotionality that does not occur exclusively in the context of a mental, psychotic or mood disorder [4]. However, the diagnosis can coexist with behavioral disorders such as Oppositional Defiant Disorder (ODD), ADHD, Conduct Disorder, and Substance Use disorders [4]. 

The different characteristics of aggression implicate different etiologies. The most effective and appropriate management of aggression can occur when there is an understanding of the etiology of the aggressive symptoms [1]. Focusing treatment to address a specific target of core symptoms can better guide treatment decisions [1]. For example, a patient with an agitated depression may respond well to an SSRI [1]. The adolescent with new onset psychosis, however, may do better with an atypical anti-psychotic [1]. The treatment plan should be created after close consideration of the risks and benefits of intervention possibilities for each particular patient. Parents need to understand the risk for self-harm, harm to others, and possible destruction of property that can occur with aggressive episodes. Likewise, they should understand the significant side-effects of medications that are being considered. In formulating a treatment plan, it is important to consider the use of all treatment modalities. There are effective non-psychopharmacological interventions to treat aggression, as well as other non-antiepileptic drug (AED) medications which have been proven efficacious. Due to the scope of this article, the discussion will be limited to the role of AEDs in the treatment of aggressive symptoms.

**Table 1:** Differentiation of aggression etiologies by clinical characteristics [1].

Outbursts Arising Out of Affective States	Outbursts Arising from Cognitive States
**Brief Impulsive Outbursts / Mild to Moderate Intensity:** Attention Deficit Hyperactivity Disorder Impulse Control Disorders **Intermediate Duration / Moderate to severe Intensity:** Major Depressive Disorder Acute Stress Disorder PTSD – Exposure to Abuse/Violence Panic and other Anxiety Disorders Adjustment Disorder with Disturbance of Conduct Borderline Personality Disorder **Prolonged Outbursts / Severe Intensity:** Bipolar Spectrum Disorder- Mania and Mixed Mania Temper Dysregulation Disorder with Dysphoria	**Outbursts Arising due Cognitive Disturbances/ confusion:** Mental Retardation Pervasive Developmental Disorders Psychotic Spectrum Disorder Delirium **Outburst During Acute Intoxication or Withdrawal:** Substance Related Disorders **Outbursts Premeditated to Achieve Desired Effect or Goal:** Conduct Disorder Antisocial Personality Disorder Narcissistic Personality Disorder

## 2. Why AEDs?

Although the atypical antipsychotics are very effective and used fairly commonly in the treatment of pediatric aggression, there is concern about their use in the pediatric population due to the incidence of metabolic abnormalities, diabetes mellitus, weight gain, insulin resistance, hyperlipidemia, neuroleptic malignant syndrome, dystonias and other extra-pyramidal reactions, including possible tardive dyskinesia [1]. Newer AEDs have improved side effect profiles and have shown some efficacy in adults for treating aggressive symptoms. AEDs may represent a more tolerable long-term treatment for aggression in children and adolescents [1]. In addition, despite the efficacy of atypical anti-psychotics, there are patients who do not respond to monotherapy. A possible augmentation strategy can include combined treatment with an AED.

## 3. Methods and Limitations

There is a limited knowledge base of well-designed, randomized, double-blind, placebo-controlled clinical trials in children and adolescents addressing the use of AEDs for aggression and/or mood lability. One of the greatest difficulties in assessing the literature is that most studies of aggression do not differentiate the etiology of aggression by diagnosis. Current diagnostic construct and outcome measures of “prolonged severe aggressive episodes” vary in clinical trials. The two most common are Bipolar Spectrum Disorders and Oppositional Defiant Disorder/Conduct Disorder with ADHD; with the former often measured using the Young Mania Rating Scale (YMRS) as an outcome measure and the later often measured using the modified overt aggression scale (MOAS). 

For this review, the Medline database was searched for articles on aggression or bipolar disorder (especially pediatric) for each of the available AEDs. Search terms included “aggression”, “bipolar disorder”, “pediatric” and “anti-epileptic drugs”. Uncontrolled studies and chart reviews were excluded where controlled clinical trials were available. Existing data were first reviewed and then synthesized clinically to determine current best evidence-based treatment guidelines. 

## 4. Results and Dissussion

AED’s have diverse mechanisms of action. The principal mechanism can be conceptualized as belonging to one of three main groups based on their predominant neurochemical effect: blockage of voltage-gated ion channels (Na and Ca), enhancement of GABA-ergic inhibition, and reduction of glutamatergic excitation [5]. AED’s may exert their action through one or more of these three mechanistic classes. For example, AED’s that work by primarily enhancing GABA-mediated transmission include tiagabine and gabapentin while those that block voltage-gated ion channels include carbamazepine, oxcarbazepine, phenytoin and lamotrigine. 

Antiepileptic drugs with higher risk of paradoxically aggravating seizures are more likely to participate in only one or two known mechanisms of action: either the blockade of voltage-gated sodium channels or facilitation of increased GABA-mediated transmission. Antiepileptic drugs that are less likely to aggravate seizures have multiple mechanisms of action identified [6]. It seems from our review, that in a similar manner, AEDs that have multiple mechanisms of action are more effective in the treatment of aggression (as with seizures) than the AEDs that have only one or two mechanisms of action, and that the latter are more likely to cause paradoxical worsening of aggression. 

### 4.1. Valproate

Of all the AEDs, the most evidence-based data currently exists for the use of valproate in the treatment of aggression. Evidence-based data has supported the efficacy of vaplroate in reducing symptoms of bipolar disorder in adults [7,8,9,10]. In adults, valproate has shown comparable efficacy to lithium [11] and superior efficacy to carbamazepine in the treatment of pure mania [12]. It is better than lithium for mania mixed with depression [12], and its efficacy is not well-established for acute bipolar depression. For prophylaxis, valproate appears to be better at preventing manic versus depressive breakthrough, but is likely inferior to lithium at preventing suicide [13]. There is an approximately 80% relapse rate with lithium or valproate monotherapy [14].

The available data for the use of valproate in youth with bipolar disorder is more conflicting. A retrospective chart review of 15 children and adolescents with bipolar disorder aged 4-18 indicated a 53% response rate after treatment with valproate for greater than one year [15]. However, a recent double-blind trial of extended-release valproate in children and adolescents aged 10-17 with bipolar disorder found that the drug did not differ from placebo after a 4-week treatment period [16].

A 12-week open-label trial of valproate in bipolar offspring at high risk for developing the disorder showed a 71% response rate to treatment based on improved scores on the Overt Aggression Scale [17]. In contrast to these results, a double-blind trial comparing valproate to placebo in youth at high risk for developing bipolar disorder found no difference between the treatment and control groups [18]. Further controlled trials are clearly necessary to evaluate the efficacy of valproate in this population.

Valproate has also been widely used in the treatment of aggression. A small study by Donovan and colleagues [19] followed 10 disruptive adolescents who met criteria for explosive mood disorder and were given open-label valproate for five consecutive weeks. All showed significant improvement and were either in remission or close to remission by the end of the study. In a later double-blind crossover trial of youth with explosive temper and mood lability, an 86% response rate to valproate was found compared to a 25% response rate for placebo [20]. While this study excluded patients with the diagnosis of bipolar disorder, many of the target symptoms in the study overlapped with those used in pediatric bipolar studies. 

Additional double-blind trials have supported the efficacy of valproate in treating impulse control amongst youth with conduct disorder [21]. Steiner *et al.* [21] compared high dose valproate to low dose valproate and monitored global severity in youth with conduct disorder. The patients receiving high dose valproate had greater improvements in their global severity rating score without a significant difference in tolerability to the valproate. Preliminary data also suggests that when valproate is used as an adjunctive treatment, it reduces aggression in children with co-morbid ADHD and disruptive behavior disorders who were refractory to stimulant monotherapy [22]. 

While double blind trials have supported its efficacy in treating irritability in children and adolescents with Autism Spectrum disorders [23], a double-blind trial evaluating valproate for aggression in youth with Pervasive Developmental disorders did not yield significant differences from placebo [24]. These results may be attributed to low power [24]. 

McVearry, *et al.* [25] recently described an increased risk of impaired cognitive function at 3 years of age in children who had in-utero exposure to valproate, as compared with other commonly used AEDs (lamotrigine and carbamazepine). Bromley, *et al*. [26] also found delayed early development in children with *in-utero* exposure to valproate. This finding supports a recommendation that valproate should not be used as a first-choice drug in women of childbearing potential.

### 4.2. Carbamazepine

In adults, carbamazepine has well established efficacy in treating aggressive symptoms in a variety of psychiatric, cognitive and neurological disorders. In the few studies using pediatric populations, results have been mixed, with larger scale trials failing to find statistically significant improvements in aggression.

In a pilot study of carbamazepine in hospitalized aggressive and explosive children with a diagnosis of conduct disorder, significant declines in aggressiveness and explosiveness were demonstrated [27]. However, in a larger double blind placebo controlled study using a similar population of aggressive children with conduct disorder, carbamazepine showed no superiority over placebo [28]. In hospitalized pre-adolescents with bipolar disorder, a recent chart review found both lithium and diavalproex to be more efficacious than carbamazepine by the second week of hospitalization [29]. 

Recently, controlled trials have explored the use of carbazepine in treating impulsive behavior in children and adults, with conflicting results in the varied populations. A double blind, placebo controlled trial found it to be an effective treatment for impulsive aggression in adult males, however, its treatment effect was slightly delayed when compared to valproate and phenytoin [30]. In a prospective randomized controlled study comparing clonidine to carbamazepine in the treatment of ADHD in children, clonidine was found to be more effective in treating impulsivity and hyperactivity [31]. Currently, carbamazepine has FDA Black Box warnings for aplastic anemia and agranulocytosis (both have an incidence of 1 in 100,000).

### 4.3. Oxcarbazepine

There is limited data regarding the psychiatric use of oxcarbazepine in children and adolescents. Two studies in the treatment of adult bipolar disorder have been promising. In a double-blind, placebo-controlled trial of oxcarbazepine in patients with impulsive aggression and no known psychiatric comorbidities, analyses showed consistent evidence of benefit from oxcarbazepine compared with placebo [32]. 

In a double-blind randomized controlled trial of oxcarbazepine *vs.* placebo in children 7 to 18 years of age with Bipolar I disorder, either manic or mixed, oxcarbazepine did not significantly improve YMRS scores at endpoint compared with placebo [29]. Dizziness, nausea, somnolence, diplopia, fatigue, and rash were each reported in at least 5% of the patients in the oxcarbazepine group with an incidence at least twice that found in the placebo group [33]. The majority of adverse events was mild to moderate and occurred during the titration period [33]. Eleven patients (19%) in the oxcarbazepine group discontinued the study because of adverse events, compared with two (4%) in the placebo group [33].

Fairly recently, the FDA released a warning of possible serious adverse dermatological reactions, including Stevens Johnson syndrome and toxic epidermal necrolysis, as well as multi-organ hypersensitivity reactions in children and adults treated with oxcarbazepine. There is a need for better efficacy and tolerability data before oxcarbazepine can be considered a first line anti-aggressive agent for children and adolescents. The advantages to using oxcarbazepine are that it does not require elaborate lab testing and has less associated weight gain than valproate [1].

### 4.4. Phenytoin

Prior to the 1990s, studies of the effects of phenytoin on aggression produced conflicting results because of failure to use objective measures of aggression across studies, lack of rigorous inclusion and exclusion criteria for selecting subjects, and non-differentiation between various types of aggression [34]. However, more recent double-blind, placebo-controlled crossover trials have indicated that phenytoin significantly reduces episodes of impulsive aggression in adults [34,35]. Controlled trials of phenytoin for adult patients with bipolar disorder suggest that the drug is effective in treating mania [36] and has prophylactic effects when used as an add-on medication for bipolar patients who had been stable for at least 1 month [37,38]. Unfortunately, no studies to date have looked at the effects of phenytoin on bipolar disorder or aggression in children.

### 4.5. Lamotrigine

Lamotrigine was approved in 2003 for maintenance therapy of adults with Bipolar I Disorder. It has a recommended slow titration schedule due to the risk of Stevens Johnson Syndrome, and for this reason is not recommended for the treatment of acute aggression. Some studies indicate a possible paradoxical increase in aggression with some patients. A recent open-label trial of the drug followed 46 pediatric bipolar patients for a duration of 14 weeks [39]. The response rates for manic and depressive symptoms were 72% and 82%, respectively, and the remission rate was 56%. An older open-label study also yielded positive results, suggesting that lamotrigine is effective either as an adjunctive treatment or as monotherapy in treating pediatric depression, mania, and aggression [40]. A double-blind placebo-controlled trial is currently underway to assess the efficacy of lamotrigine as an add-on treatment for children and adolescents with bipolar disorder.

In adult double-blind studies, it has been shown to be particularly useful in prevention of relapse into depression. In placebo-controlled studies of bipolar depression by Calabrese *et al.*, secondary outcomes for lamotrigine monotherapy at 200 mg were significant when compared to monotherapy at 50 mg and placebo [41]. In a comparison of lamotrigine to placebo in Bipolar I and II depressed patients, no overall difference was found. However, a separate analysis of the Bipolar I subgroup found better response on lamotrigine than placebo [41]. While lamotrigine may be effective for prophylaxis of bipolar depression, it would not be recommended for acute aggression due to the necessity for a slow titration. 

### 4.6. Topiramate

Topiramate has been shown to be an effective treatment for aggression in adult populations with borderline personality disorders in double blind placebo controlled trials, with improvements maintained at 18 month follow up [42,43,44]. Based on 14 open label studies, the drug had an overall 58% response rate in 279 adult patients with Bipolar disorder [1]. In double-blind placebo-controlled studies, Yatham *et al.* [45] and Calabrese *et al.* [46] reported that topiramate had no difference in primary outcomes for acute mania, but did show better efficacy on secondary outcomes. 

Pediatric studies using small samples of children with Autism Spectrum disorders have not found topiramate to be an effective agent in reducing self-injurious behaviors. In one such study using a sample of 5 severely impaired boys with Autism Spectrum disorders aged 9-13, topiramate was not found to be substantially effective over a treatment period ranging from 10-33 weeks [47]. In a sample of 10 children and adolescents aged 8-19 with Autism Spectrum disorder who were on antipsychotic medication, topiramate (added on to counteract neuroleptic-induced weight gain) caused “behavioral adverse effects” in three patients, requiring rapid withdrawal of this medication [48]. The authors concluded that topiramate should be used with caution in Autism Spectrum disorders [48]. Additionally, topiramate, among the newer AEDs, has the greatest risk for cognitive impairment [49].

In pediatric populations, chart reviews have suggested the possible efficacy of topiramate as an adjunctive treatment for bipolar disorder, however this has not been confirmed in controlled trials. In a retrospective chart review looking at 26 patients with a diagnosis of bipolar disorder between the ages of 10-18, topiramate was found to be a well tolerated and effective adjunctive treatment [50]. However, a pilot double blind placebo controlled trial examining the efficacy of topiramate for mania in 56 children and adolescents with bipolar disorder was discontinued when adult mania trials failed to show efficacy [51]. In another more recent retrospective chart review of 25 hospitalized children and adolescents with bipolar disorders receiving topiramate as an adjunctive treatment, topiramate was found to be effective for acute, manic, mixed, or depressive episodes [52]. 

Perhaps the greatest demonstrated advantage to the use of topiramate compared with other AED’s is the associated weight loss. In two open label trials looking at topiramate as monotherapy [53,54] or as an adjunctive treatment [53], both found significant reductions in weight or reduced weight gain. 

### 4.7. Levetiracetam

Levetiracetam has been explored as a treatment for aggression and impulsivity in a number of different adult populations, with mixed effectiveness outcomes. In a small open label on-off-on study design of adjunctive levetiracetam therapy with 10 acutely manic adults with Bipolar I disorder, improvements were found during the “on” phase [55]. However, a larger 10 week double blind placebo controlled trial of 40 outpatients with clinically significant impulsive aggression, did not find levetiracetam to be effective in reducing patient aggression [56]. 

In the pediatric population, levetiracetam has been studied as a potential treatment for the impulsivity, aggression and mood lability associated with autism. Earlier open label trials using smaller samples indicated potential efficacy of levetiracetam as a treatment for aggression in autistic populations, with these findings not being confirmed in larger controlled trials. An open label study of the effectiveness of levetiracetam in a sample of 10 autistic boys aged 4-10 found significant reductions in inattention, hyperkinesis and impulsivity [57]. In a larger 10 week double blind placebo controlled study of 25 autistic patients aged 5-17, findings of the effectiveness of levetiracetam in treating the behavioral disturbances associated with autism was not confirmed [58].

There have been reports of behavioral changes, mostly negative (including aggression), in patients taking levetiracetam [59]. Due to the lack of good evidence base in supporting the efficacy of levetiracetam for aggression, and reports of worsening aggression, we do not support the use of this medication as a first-line treatment of aggression.

### 4.8. Zonisamide

There is very little data available regarding the use of zonisamide for aggression. Thus far, it has primarily been utilized in the treatment of bipolar depression. In an open-label study of bipolar and schizoaffective manic adults, 71% showed significant global improvement on zonisamide [60]. Another preliminary study of zonisamide in 10 patients with bipolar depression found significant clinical global improvement scores and reduction in depressive symptoms [61]. However, there was no significant effect of the drug on improving mania scores in these patients. The most recent study of zonisamide for bipolar depression found the drug to have modest effectiveness and a low switch rate to mania, but the authors did not recommend its use as a first line treatment due to a high side-effect profile [62]. To date, zonisamide has not been studied for the psychiatric treatment of children.

### 4.9. Gabapentin

Gabapentin is generally not useful for aggression. An exception is that it may prove helpful in select cases where anxiety or pain is leading to aggression [63]. 

In a review on the use of gabapentin in mood disorders in adult populations, 40 open-label studies and four controlled trials were identified [64]. Based on the open-label studies and two of the controlled trials, the authors concluded that gabapentin may have a possible benefit as an adjunctive agent in treating refractory and co-morbid bipolar disordered patients [64]. However, we consider this to be an overly optimistic conclusion, given that the two trials that were positive had small sample sizes and although the findings were presented at conferences/meetings, we were unable to find these published in the peer-reviewed literature. The two controlled trails that did not show evidence of gabapentin’s superiority to placebo have been published in peer-reviewed journals [63,65]. 

In the pediatric literature, gabapentin has been noted to worsen and even induce aggression in certain populations [66]. It has also been reported to cause aggressive and oppositional behavior in pediatric patients with seizures [67,68].

### 4.10. Tiagabine

Tiagabine is a GABA-reuptake inhibitor which requires slow titration due to the significant adverse effects noted in open label studies [1]. Due to the purported relationship between GABA and aggressive behavior in humans, a recent study looked at the effect of acute tiagabine administration on the aggressive response rate of 10 adult male parolees, with findings indicating that tiagabine decreased aggressive responding in some domains of laboratory response tasks [69].

There have been a number of open label trials testing the effectiveness of tiagabine in treating adult patients with bipolar disorder. In an open label trial of eight acutely manic adult patients with a diagnosis of Bipolar I disorder receiving either tiagabine monotherapy (n = 2) or adjunctive tiagabine (n = 6), there were no reductions in mania over the 2 week observation period, and severe side effects such as nausea, vomiting and tonic-clonic seizure occurred in two patients [70]. 

Adult open label trials of tiagabine as an adjunctive treatment for refractory bipolar disorder have yielded mixed results. In an open label trial of tiagabine as an add-on treatment for 17 treatment refractory adult bipolar patients, there was a 23% response rate with 77% showing no change or worsening symptoms [71]. Another open label trial of tiagabine as an adjunctive treatment for 22 refractory bipolar patients found a 36% response rate [72]. However, 64% of patients in this study also had to discontinue the medication secondary to adverse effects [72].

Recently, after more than 30 post-marketing reports of new onset seizures and status epilepticus in patients with no history of epilepsy, the FDA has issued a warning of the incidence of de-novo seizures in patients treated off-label with tiagabine. No pediatric trials were identified from the literature for this review.

### 4.11. Head to Head Comparisons

There have been few studies comparing the efficacy of AEDs with each other in the treatment of pediatric aggression. MacMillan and colleagues [73] compared divalproex, oxcarbazepine, and gabapentin in youth with aggression and found that although the percentage of responders at 6 months did not differ significantly between groups, there were significant differences in discontinuation rates. Gabapentin and oxcarbazepine had higher rates of discontinuation due to worsening of aggression, 13.3% and 14.3% respectively, when compared to divalproex (0.0%). In addition, more patients in the gabapentin group (40%) were worse overall at the end of the observation than in either the divalproex (13.8%), or oxcarbazepine (19%) groups. In a study by Kowatch *et al.* [73,74] comparing monotherapy with valproate, lithium, or carbamazepine in children and adolescents with bipolar disorder, response rates were 53%, 38%, and 38% respectively. Results from these studies implicate valproate as the most efficacious AED in the treatment of pediatric aggression.

### 4.12. Combined Pharmacotherapy

The review of this literature seems to suggest greater efficacy with combination treatment. In an open-label study of combined treatment with divalproex and lithium in pediatric patients with bipolar disorder, Findling *et al.* [18] found that 47% met the stronger *a priori* criteria for remission. Kowatch *et al.* [75] conducted a 16 week extension phase of monotherapy treatment with valproate, carbamazepine, or lithium. Among those patients that did not respond to monotherapy, 80% responded to a combination of two mood stabilizers.

In a study by Findling *et al.* [76], 139 youth aged 5-17 with Bipolar I or II Disorder were initially treated with lithium plus valproate for approximately 11 weeks. Patients meeting remission criteria for four consecutive weeks were then randomized in a double-blind fashion to treatment with either lithium or valproate for up to 76 weeks. The lithium and valproate treatment groups did not differ in survival time until emerging symptoms of relapse or in survival time until discontinuation for any reason.

In an open-label prospective trial of risperidone in combination with lithium or divalproex sodium in pediatric mania, 37 subjects aged 5 to 18 with DSM IV current mixed or manic episode were sequentially assigned to either valproate plus risperidone or lithium plus risperidone in a 6-month, prospective open-label trial [77]. Effect sizes based on change of YMRS scores from baseline were 4.36 for valproate plus risperidone and 2.82 for lithium plus risperidone. Response rates were 80% for valproate plus risperidone and 82.4% for lithium plus risperidone [77]. Both combination treatments were well tolerated [77]. There were no significant group differences in safety or tolerability and no serious adverse events during the 6-month trial [77]. 

In a retrospective chart review, DelBello *et al.* [78] found that when divalproex was combined with quetiapine in juvenile patients with Bipolar Disorder, significantly more reached remission criteria as compared to divalproex alone. These patients, however, also had increased weight gain.

Gonzalez-Heydrich *et al.* conducted a retrospective chart review of hepatic enzyme levels during treatment with olanzapine, divalproex, or a combination of olanzapine and divalproex treatment. Patients on combined olanzapine and divalproex therapy had an increased risk of elevated hepatic enzymes compared to those on divalproex or olanzapine as sole mood stabilizers, including two of 12 patients with elevations greater than three times the normal range [79]. Combination treatment was discontinued due to pancreatitis in one child and steatohepatitis in another.

Thus it seems that combination treatment of an AED with another AED, or lithium, or an atypical antipsychotic, is more efficacious than treatment with one AED alone. However, the incidence of side effects is also higher.

## 5. Conclusion: Summary of the Efficacy of AEDs in Pediatric Aggression

From the available data regarding the efficacy of treating pediatric aggression with AEDs, two trends were observed. First, medications with predominately GABA-ergic mechanisms of action have not been found to be effective in treating aggression, as demonstrated by the data from trials of gabapentin and tiagabine, with results demonstrating little efficacy or exacerbation of symptomatolgy [1]. It is not known why a predominate effect of increasing GABA activity can frequently lead to a paradoxical increase in aggression. That similar effects have been seen with such GABA agonists as ethanol and benzodiazepines, implies that some degree of facilitation of impulsive behavior is occurring. There is some evidence that medications which work by stabilizing the sodium and calcium channels may be helpful, indicated by valproate, the AED with the most evidence based data [1]. Secondly, agents with multiple mechanisms of action may be more effective. This second trend may explain an apparent contradiction that enhanced GABA activity, when accompanied by additional antiepileptic mechanisms, does not lead to the same high rate of paradoxical worsening of aggression. For example, valproic acid’s potential enhancement of GABA mechanisms at higher doses, together with its actions on sodium and calcium channels, may provide a possible explanation for the more robust improvement seen with valproate at higher doses compared to that seen for carbamazepine [1]. This leads to the potentially testable hypothesis that adding a pure GABA enhancing agent to an agent such as carbamazepine may augment its effect. Finally, medications like topiramate which decrease glutaminergic transmission tended to have more cognitive adverse effects [1].

While there is a limited knowledge base of well-designed, randomized, double-blind, placebo-controlled clinical trials in children and adolescents addressing the use of AEDs for aggression and/or mood lability, currently, the strongest evidence exists for valproate. It has the added benefit of being loaded quickly at an approximate 20-30 mg/kg daily dose with a target steady state blood level between 80 and 125 mcg/mL [1]. Common adverse reactions include weight gain, liver function and platelet abnormalities [1]. Rare cases of hepatic necrosis and pancreatitis must be watched for; and drug blood levels, serum amylase, plasma ammonia, liver function tests, and platelets need to be monitored [1]. Patients doing poorly on valproate should first have their blood level checked and dosage optimized as patients receiving high dose valproate had greater improvements in their global severity rating score [21]. Due to the evidence for early cognitive delays in children exposed to valproate in-utero [25,26], we do not recommend valproate as a first-line agent for the treatment of aggression in females of child-bearing age.

When monotherapy is not sufficient, combining an AED with either lithium or an atypical anti-psychotic can result in better efficacy, but may also lead to more weight gain, metabolic side-effects, and liver toxicity [1]. Also, a more careful differential diagnosis of aggression and identification of patients that might respond to ADHD or antidepressant medication is warranted. When using combined treatments, more careful monitoring and discussions of risks and benefits is warranted [1]

Our review of the literature reveals that most of the AEDs show significant improvement in pediatric aggression, however, not a clear improvement in pediatric bipolar disorder. The definition of pediatric bipolar disorder is currently controversial, and most patients currently diagnosed with pediatric bipolar disorder do not actually meet strict DSM-IV definitional criteria. The authors of the next edition of the DSM, the DSM-V, propose to deal with this nosological challenge by introducing a new diagnosis, “Temper Dysregulation Disorder with Dysphoria” [80] to include most cases currently being diagnosed as pediatric bipolar disorder. We feel that this change will be a step in the right direction, given what we have found in our literature review with these AED’s: that pediatric aggression is a phenotype that is distinct from pediatric bipolar disorder at least as far as treatment is concerned. However, it remains to be seen if this change in DSM terminology will actually occur.

It is worth mentioning that although all AEDs now carry a controversial FDA mandated warning regarding a potential increase in suicidality [1], there has been recent evidence that AEDs do not increase the risk of suicide attempts in patients with bipolar disorder compared to untreated patients, and that the use of AEDs actually decreases the rates of suicide attempts relative to untreated patients and relative to the patients’ pretreatment levels [81].

Finally, the authors would also like to make a brief mention of Lithium, which, although beyond the scope of this review on account of it not being an AED, has been extensively used for impulsive aggression and especially bipolar disorder and aggression stemming from mania. It has been shown to be better than placebo in three of five placebo controlled studies [82,83].

**Table 2:** Summary of available evidence on AEDs for pediatric aggression (studies in adults are not included).

AED	PROS and Positive Results	CONS and Negative Results
VALPROATE Most evidence based data of all the AEDs	Double blind trials support its efficacy in treating impulse control amongst youth with conduct disorder [21]. 86% response rate (compared to 25% for placebo) in a double blind crossover trial of youth with explosive temper and mood lability [20]. 71% response rate in bipolar offspring at high risk of developing the disorder in an open label trial [17]. 53% response rate in children with bipolar disorder after treatment with Valproate for greater than 1 year based on a retrospective chart review [15]. Some double blind placebo controlled trials have supported its efficacy in treating irritability (not aggression) in children and adolescents with Autism Spectrum Disorders [23]. Preliminary data suggests that as an adjunctive treatment, it reduces aggression in children with co-morbid ADHD and disruptive behavior disorders who were refractory to stimulant monotherapy [22].	No difference to placebo in the treatment of Bipolar Disorder [16] and also those at high risk for developing Bipolar Disorder [18] in double blind placebo controlled trials. No significant difference from placebo in a double blind placebo controlled trial evaluating efficacy in treating aggression in Pervasive Developmental Disorders [24] (result may be due to low power). Increased risk of impaired cognitive function in children with in utero exposure to valproate compared to other commonly used AEDs [25].
CARBAMAZEPINE In adults, carbamazepine has well established efficacy in treating aggression, but in the few studies using pediatric populations, results have been mixed.	Significant declines in aggressiveness and explosiveness in aggressive and explosive children with conduct disorder were demonstrated in an open label trial [27].	Superiority of carbamazepine over placebo in aggressive and explosive children with conduct disorder not confirmed by double blind placebo controlled trials [28]. Less effective than clonidine in treating impulsivity and hyperactivity in children with ADHD based on results from a randomized controlled trial [31] FDA Black Box warnings for aplastic anemia and angranulocytosis (both have an incidence of 1 in 100,000).
OXCARBAZEPINE Limited data regarding its use in pediatric population. Two studies for treating adult bipolar disorder were promising.	It does not require elaborate lab testing. Has less associated weight gain than valproate.	No superiority to placebo for children with Bipolar I, manic or mixed, found in a double-blind randomized controlled trial. FDA Warning of possible serious adverse dermatological reactions including Stevens Johnson syndrome and toxic epidermal necrolysis and multi-organ hypersensitivity reactions
PHENYTOIN	Although controlled trials have shown efficacy in treating aggression and mania in adults, no studies have looked at the effects of phenytoin on bipolar disorder or aggression in children	
LAMOTRIGINE	In a recent open label study of pediatric bipolar patients a response rate of 72% was found for manic symptoms and 82% for depressive symptoms. The remission rate was 56% [39].	Not recommended for acute aggression due to the slow titration requirement.
TOPIRAMATE	There is associated weight loss – 2 open label trials showed significant reductions in weight or reduced weight gain [53,54]. Chart reviews [50,52] have suggested the possible efficacy of topirimate as an adjunctive treatment for pediatric bipolar disorder, however this has not been confirmed in controlled trials.	A double blind placebo controlled trial of topirimate for mania in children with bipolar disorder was discontinued when adult trials failed to find efficacy [51]. Has not been found to be an effective treatment for reducing self injurious behaviors in children with Autism Spectrum Disorders [47,48]. Among the newer AEDs, has the greatest risk for cognitive impairment [49].
LEVETIRACETAM	Earlier, small open label trials suggested efficacy of levetiracetam as a treatment for aggression in pediatric populations [57], but this was not confirmed in controlled trials.	Double blind placebo controlled trials have not found levetiracetam to be effective in treating the behavioral disturbances associated with autism in the pediatric population [58]. There have been reports of negative behavioral changes, including aggression, in patients taking levetiracetam [59].
ZONISAMIDE	Not studied for the psychiatric treatment of children	
GABAPENTIN	May prove helpful in select cases, where anxiety or pain is leading to aggression [63].	Noted to worsen and even induce aggression in certain pediatric populations [66]. It has been reported to cause aggressive and oppositional behavior in pediatric patients with seizures [67,68].
TIAGABINE No pediatric trials		FDA has issued a warning of the incidence of de-novo seizures in patients treated with off label tiagabine.
